# Synthesis of biogenic silver nanoparticles using *Althaea officinalis* as reducing agent: evaluation of toxicity and ecotoxicity

**DOI:** 10.1038/s41598-018-30317-9

**Published:** 2018-08-17

**Authors:** Diogo Torres Rheder, Mariana Guilger, Natália Bilesky-José, Taís Germano-Costa, Tatiane Pasquoto-Stigliani, Tatiane Balbo Batista Gallep, Renato Grillo, Cleoni dos Santos Carvalho, Leonardo Fernandes Fraceto, Renata Lima

**Affiliations:** 1 Federal University of São Carlos, Sorocaba campus, Rodovia João Leme dos Santos, km 110, 18052-780 Sorocaba, Brazil; 2 Labiton - Laboratory of Bioactivity Assessment and Toxicology of Nanomaterials, University of Sorocaba, Rodovia Raposo Tavares, km 92, 18023-000 Sorocaba, São Paulo Brazil; 3 São Paulo State University (UNESP), Department of Physics and Chemistry, School of Engineering, Avenida Brasil, 56, 15385-000 Ilha Solteira, SP Brazil; 4 São Paulo State University (UNESP), Institute of Science and Technology of Sorocaba, Laboratory of Environmental Nanotechnology, Av. Três de Março, 511, 18087-180 Sorocaba, São Paulo Brazil

## Abstract

Silver nanoparticles (AgNPs) are known mainly because of their bactericidal properties. Among the different types of synthesis, there is the biogenic synthesis, which allows the synergy between the nanocomposites and substances from the organism employed for the synthesis. This study describes the synthesis of AgNPs using infusion of roots (AgNpR) and extract (AgNpE) of the plant *Althaea officinalis*. After the synthesis through reduction of silver nitrate with compounds of *A*. *officinalis*, physico-chemical analyzes were performed by UV-Vis spectroscopy, nanoparticles tracking analysis (NTA), dynamic light scattering (DLS) and scanning electron microscopy (SEM). Toxicity was evaluated through *Allium cepa* assay, comet test with cell lines, cell viability by mitochondrial activity and image cytometry and minimal inhibitory concentration on pathogenic microorganisms. Biochemical analyzes (CAT - catalase, GPx - glutathione peroxidase e GST - glutationa S-transferase) and genotoxicity evaluation *in vivo* on Zebrafish were also performed. AgNpE and AgNpR showed size of 157 ± 11 nm and 293 ± 12 nm, polydispersity of 0.47 ± 0.08 and 0.25 ± 0.01, and zeta potential of 20.4 ± 1.4 and 26.5 ± 1.2 mV, respectively. With regard to toxicity, the AgNpE were the most toxic when compared with AgNpR. Biochemical analyzes on fish showed increase of CAT activity in most of the organs, whereas GPx showed few changes and the activity of GST decreased. Also regarding to bactericidal activity, both nanoparticles were effective, however AgNpR showed greater activity. *Althaea officinalis* can be employed as reducing agent for the synthesis of silver nanoparticles, although it is necessary to consider its potential toxicity and ecotoxicity.

## Introduction

Nanotechnology has shown considerable potential for use in diverse areas including the textile, medical, pharmaceutical, cosmetics, agricultural, and chemical sectors^1–4^. Among the nanomaterials, metallic nanoparticles have attracted attention, especially in the medical and engineering fields, due to the unique properties that metals acquire at the nanometric scale^5^, as well as the possibility of conjugated synthesis and surface functionalization^6^, which opens up possibilities for a wide range of applications, in which silver nanoparticles are among those most extensively used, due to their proven bactericidal properties^7–13^.

There are different methods employed to synthesize nanoparticles and biogenic techniques are of particular interest since they are considered sustainable and allow synergy between the metal precursor and enzymes, proteins, polysaccharides, and other active substances derived from the reducing organism (*Althaea officinalis*, in the case of the present study)^14–16^.

Biogenic nanoparticles produced using plant extracts as reducers are among those that have received most attention^10,17–31^. Although the reduction of metal ions by plant extracts has been known since the 20^th^ century, the mechanisms of bioreduction have not been fully elucidated^32^, however some studies have indicated that it involves enzymatic action of compounds in the extract^33,34^, specially enzymes such as nitrate reductase. The biogenic synthesis of AgNPs and their application in the control of bacteria has been shown to be feasible, as demonstrated by Makarov *et al*.^20^, who obtained promising results using the technique with different plants. Benelli *et al*.^35^ highlighted the effectiveness, low cost, and simplicity of synthesizing nanoparticles using botanical extracts, and described applications of these materials for the control of insects that are pests and disease vectors in agriculture. The authors noted the importance of studying the mechanisms of action of these nanomaterials and understanding their potential toxicity towards non-target organisms. As example, Guilger *et al*.^15^ described the potential of this kind of nanoparticles for the control of an important pathogenic fungus in agriculture.

The extract of the plant *Althaea officinalis* (marshmallow) has been used for many years to treat inflammations and wounds^36^. The root is widely used due to its ability to retain water and its high content of polysaccharides that can stimulate the immune system^37^. This plant could therefore be an attractive option for use as a reducer in biogenic synthesis.

In this study two types of biogenic AgNPs were synthesized using leaf extract and infusion of roots of *Althaea officinalis* as a reducing agent and their physico-chemical characteristics were evaluated. In order to verify the possible toxicity and antimicrobial potential the assays reduction of tetrazolium through mitochondrial activity (MTT), image cytometry, comet and minimal inhibitory concentration were performed. Ecotoxicity was also evaluated through *Allium cepa* assay and comet and biochemical assays with zebrafish.

## Methods

### Biological materials

#### Cell lines

Cyto and genotoxicity assays employed the standard ATCC cell lines 3T3, HaCat, A549, V79 and HeLa, obtained from the Rio de Janeiro cell bank. The cells were first grown, then trypsinized and transferred to 6, 12, 24 or 96-well plates, depending on the assay to be performed.

#### Microbial strains

The minimum inhibitory concentration (MIC) tests employed standard strains of *Staphylococcus aureus*, *Escherichia coli*, *Pseudomonas aeruginosa*, and *Candida albicans* (ATCC numbers 25923, 25922, 10145, and 10231, respectively), as recommended for use in antimicrobial susceptibility tests^38^. The strains were supplied by Cefar (São Paulo, Brazil).

#### Fish

Juvenile *Danio rerio* (Zebrafish) (body mass = 0.5–0.9 g; total length = 2–3 cm) from the World Fish, Brazil, were acclimated to the laboratory for 10 days in holding tanks (3L) at 25 ± 1 °C, with a continuous flow of aerated, dechlorinated tap water (pH 7.0 ± 0.2; conductivity = 8.3 ± 0.3 µS; alkalinity = 23.7 ± 1.9 mg.L^−1^ as CaCO_3_; hardness = 24.5 ± 0.2 mg.L^−1^ as CaCO_3_) and in a 12D:12L (12Dark:12Light) photoperiod. The fish were fed with balanced fish food appropriate for this species (TetraMin Tropical Flakes) every 48 h. Feeding was suspended 24 h before assays. All experiments were performed in accordance with relevant guidelines and regulations. All experimental protocols were approved by the Animal Research Ethical Commission of the University of Sorocaba (Protocol 004/2012).

### Synthesis and physicochemical characterization of the nanoparticles

The nanoparticles were synthesized using *Althaea officinalis* leaf extract and dehydrated root infusion. The procedure was performed as described by Logeswari, Silambarasan and Abraham^39^, with minor modifications, mixing 20% of silver nitrate solution (0.1 mol.L^−1^) with 80% of extract or infusion solution (0.2 g.mL^−1^). The mixture was stirred in the dark for 24 h.

Firstly, physicochemical characterization of the synthesized nanoparticles was performed by UV-Vis spectroscopy, determination of the hydrodynamic diameter (nm), polydispersity index (PDI), and zeta potential by dynamic light scattering (DLS) and microelectrophoresis techniques. The analyses were performed at a fixed angle of 90° and temperature of 25 °C, using a Zetasizer Nano ZS90 system (Malvern Instruments). The nanoparticles concentration (NP mL^−1^) and size distribution were determined by nanoparticle tracking analysis (NTA), using a NanoSight LM14 instrument and NanoSight v.2.3 software. The water used to perform the ecotoxicity assays was also evaluated through the same analyses after exposing the fish to the nanoparticles, in order to identify any changes resulting from the exposure. Also, scanning electron microscopy (SEM) was performed in order to investigate the morphology and size distribution of the nanoparticles. Nanoparticles suspensions were diluted (1:50 v:v), dried in a silicon grids and analyzed by scanning electron microscopy (EVO-LS-15, Carls Zeiss). SEM was operated at 20 kV of high voltage with a spot size between 3.0–4.0 and working distance (WD) of 8.5 mm. Size distribution histogram of the AgNPs was calculated using ImageJ and GraphPad Prism 7.0 software, and approximately 300 nanoparticles were counted in each study.

### Evaluation of cell viability through mitochondrial activity (MTT)

For the evaluation of cell viability, the mitochondrial activity was determined by the reduction of MTT (4-5-dimethylthiazolyl-2)-2,5-diphenyltetrazolium bromide) salt. The cells (3T3, HaCat, A549, V79 and HeLa) were transferred to 96-well plates, at a concentration of 2 × 10^4^ cells per well, and were kept at 37 °C for 24 h, with 5% CO_2_. After adherence, the cells were exposed for 24 h to the AgNPs at decreasing concentrations (3 × 10^9^, 10^8^, 10^7^, 10^6^, 10^5^, 10^4^, 10^3^, and 10^2^ NPs.mL^−1^). The cultures were then washed with PBS, followed by addition of 100 μL of MTT solution (0,5 mg mL^−1^) to each well and incubation for 3 h at 37 °C, with 5% CO_2_. After the incubation period, the culture medium was removed and 100 μL of Dimethyl Sulfoxide (DMSO) was added to each well. The readings were performed at 570 nm using a microplate reader (Evolution 201, Thermo Scientific).

### Cell viability, apoptosis and necrosis by imaging cytometry analyses

Imaging cytometry was used to evaluate cell viability, apoptosis, and necrosis. The cells (A549 and V79) were exposed to the nanoparticles at a concentration of 3 × 10^8^ NPs.mL^−1^ for 1 h, trypsinized and centrifugated. The supernatant was discarded and the samples were prepared using a Tali™ Apoptosis Kit (Annexin V AlexaFluor® 488 and Propidium Iodide, Invitrogen), according to the manufacturer’s instructions. The readings were performed using Tali™ imaging cytometry.

### Evaluation of DNA damage by comet assay

The comet assay was adapted from Azqueta *et al*.^40^, exposing 3T3, A549, V79 and HeLa cell lines to nanoparticles at a concentration of 3 × 10^8^ NPs.mL^−1^. The cells were classified by visual scoring, using 0 to 4 categories, where 0 represents damage absence and 4 indicates the most intense damage^41^. The analyses were performed in duplicate, with observation of at least 200 randomly selected cells. The final scores were obtained by multiplying the number of cells allocated to each category by the category number. The damage index (DI) was calculated by dividing the sum of the values for each category by the total number of cells analyzed.

### Evaluation of antimicrobial potential using minimum inhibitory concentration (MIC) assays

MIC assays were performed with the *E*. *coli*, *S*. *aureus*, *C*. *albicans*, and *P*. *aeruginosa* strains grown in Mueller-Hinton broth for 24 h. After growth, 10 µL of microorganism suspensions at a concentration of 5 × 10^5^ CFU.mL^−1^ were added to 96-well plates with culture broth and decreasing concentrations of AgNpE and AgNpE (3 × 10^9^ to 10^2^ NPs.mL^−1^). The plate was incubated at 37 °C for 24 h, followed by addition of 10 μL of Resazurin (6.75 mg.mL^−1^) to each well and incubation for a further 24 h. The results were obtained by colorimetric analysis.

### Ecotoxicity evaluation by *Allium cepa* assays

Previously germinated roots of *Allium cepa* were exposed to AgNpR and AgNpE at a concentration of 3 × 10^8^ NPs.mL^−1^ during 24 h. After exposure, the roots were fixed with Carnoy’s solution (3:1 ethyl alcohol and acetic acid, v-v), washed, and submitted to acid hydrolysis with 1 mol.L^−1^ hydrochloric acid at 60 °C for 9 min. Coloration was performed by reaction with Schiff reagent for 2 h in the dark at room temperature, and the slides were prepared by separating the meristematic region, adding a drop of 2% Acetic Carmine dye and smashing to separate the cells. Analyses were performed in triplicate with observation of at least 1,500 cells. Mitotic index (MI) and alteration index (AI) were calculated employing Eqs 1 and 2, respectively.

1 $$MI=\frac{no\,of\,cells\,in\,division}{total\,number\,of\,cells}$$

2 $$DI=\frac{no\,of\,cells\,with\,chromosome\,damage}{total\,number\,of\,cells\,in\,division}$$

### Evaluation of ecotoxicity in zebrafish

#### Fish exposure and removal of biological material

The fish were separated into different aquaria (n = 15 per aquarium) and exposed to the nanoparticles at concentrations of 3 × 10^5^, 3 × 10^6^, 3 × 10^7^, and 3 × 10^8^ NPs.mL^−1^ for 72 h, after which they were anesthetized using benzocaine (250 mg.L^−1^). Animals were then sacrificed by painless medulla section (Resolution no. 1000, March 11^th^, 2012: Procedures and Methods of Euthanasia in Animals), from which 10 µL of blood were collected into tubes containing fetal bovine serum and Ethylenediaminetetraacetic acid (EDTA). The organs (gills, brain, heart, liver, skin, and muscle) were then collected and stored according to the analyses to be performed.

#### Quantification of proteins and determination of enzymatic activity

For the biochemical analysis, the organs (brain, heart, liver, skin, and muscle) collected from zebrafish were homogenized in 0.5 mL of Phosphate-Buffered Saline (PBS) buffer (pH 7.2), the mixture was centrifuged at 12,000 *g* for 20 min at 4 °C, and the supernatant was collected in tubes. This material was used immediately or stored at −80 °C for subsequent use.

Proteins were quantified using the method of Bradford^42^. The sample and the solution were applied to the plate and kept for 5 min in the absence of light, followed by reading of the absorbance at 595 nm using a Tecan Infinite M200 Pro plate reader.

In order to investigate the possible use of zebrafish in biomonitoring, it was evaluated the enzymatic activity of catalase (CAT), glutathione peroxidase (GPx), and glutathione S-transferase (GST), which have been linked to the redox cycle and detoxification processes.

The CAT activity was determined as described by Aebi^43^, with the sample dispersed in reaction buffer (1 M Tris-HCl and 5 mmol.L^−1^ EDTA) in a quartz cuvette. The absorbance was measured at 240 nm, at 15 s intervals during 2 min, using a PerkinElmer Lambda 35 spectrophotometer.

The GPx activity was evaluated as described by Beutler^44^. A 20 μL aliquot of the supernatant of the protein sample was pipetted into a 96-well plate, followed by addition of 140 μL of reaction medium (2 mmol.L^−1^ sodium azide, 0.2 mmol.L^−1^ NADPH, 2 mmol.L^−1^ GSH, 1 U.mL^−1^ glutathione reductase, and 14.45 mL of 0.1 mol.L^−1^ PBS, at pH 7). After 2 min, 40 μL of hydrogen peroxide solution was added and the absorbance was measured at 340 nm using the Tecan Infinite M200 Pro microplate reader.

The GST activity was evaluated according to Keen *et al*.^45^, with modifications. A 20 μL aliquot of the protein sample was added to a well of the microplate, followed by addition of 180 μL of reaction medium (1.5 mmol.L^−1^ GSH, 2 mmol.L^−1^ CDNB (1-chloro-2,4-dinitrobenzene), and 0.1 mol.L^−1^ PBS, at pH 6.5). The absorbance was measured at 340 nm. The activity was calculated using the molar extinction coefficient for CDNB (9.6 mmol^*−*1^.cm^−1^).

These biomarkers were selected because they act in the main enzymatic defenses of the organisms and are the most sensitive^46^.

#### Evaluation of DNA damage using comet assay *in vivo*

For the performance of the *in vivo* comet assay, blood and gills from zebrafish were collected into tubes containing fetal bovine serum and EDTA, homogenized and used according to the method previously described for *in vitro* comet assay.

#### Statistical analyses

Statistical analysis was performed using one-way analysis of variance (ANOVA) and the Tukey-Kramer test, with a significance level of p < 0.05, by GraphPad Prism 7.0 software.

### Availability of data and materials

The datasets used and/or analyzed during the current study are available from the corresponding author on reasonable request.

## Results and Discussion

### Physicochemical characterization of the nanoparticles

The nanoparticles were characterized immediately after the synthesis and after fish exposure assays. The first technique performed was UV-Vis spectroscopy in order to confirm the synthesis. Bands of 384 and 380 nm were obtained for AgNpE and AgNpR, respectively (data not shown). These bands indicate the surface plasmon resonance of silver nanoparticle (Ag^o^), confirming that a successful synthesis was performed^47^.

**Figure 1:**
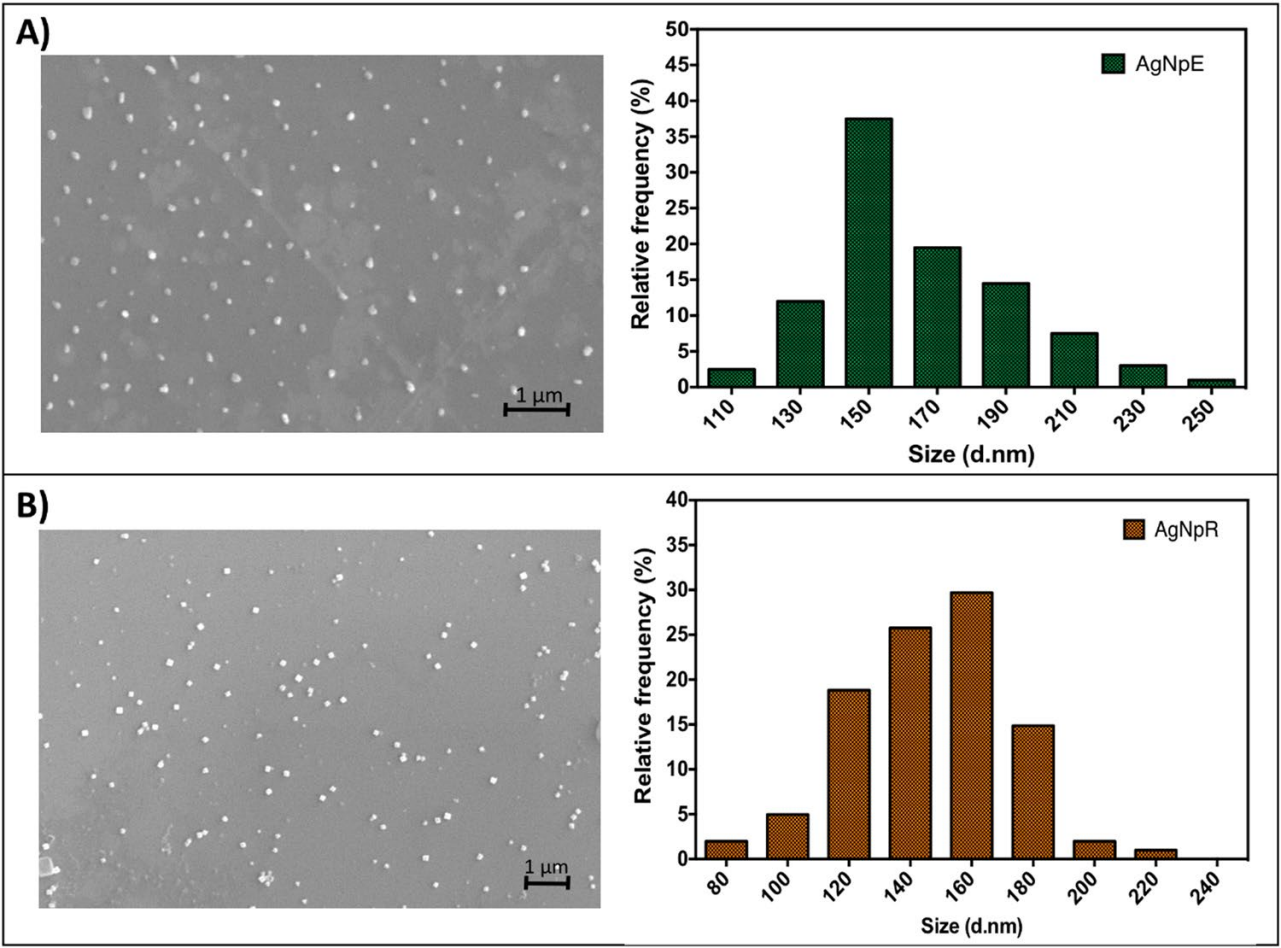
Size histograms of biogenic silver nanoparticles measured by Scanning Electron Microscopy (SEM): (**A**) AgNpE and (**B**) AgNpR.

The initial analyses showed that the AgNpR were larger and more stable than the AgNpE, while the concentration of AgNpE was slightly larger than the AgNpR, as shown by NTA (Table 1) and SEM images (Fig. 1). Therefore, it did not influence in our studies since the concentrations of the nanoparticles in all subsequent assays were adjusted to 3 × 10^10^ NPs.mL^−1^ in aqueous medium (corresponding to 3.4 mg.L^−1^). Durán *et al*.^48^ and Chandran *et al*.^49^ also obtained biogenic nanoparticles with similar sizes ranging between 130 and 300 nm (measured by DLS) and zeta potentials close to 30 mV, corroborating with our studies.

**Table 1:** Physicochemical characterization of the nanoparticles using DLS and NTA.

Technique	Nanoparticle	Size (nm)	Polydispersity	Zeta potential (mV)	Concentration (NPs/mL)
DLS	AgNpE	157 ± 11	0.477 ± 0.08	20 ± 1	—
	AgNpR	293 ± 12	0.254 ± 0.03	26 ± 1	—
NTA	AgNpE	131 ± 5	—	—	7.2 × 10^10^
	AgNpR	227 ± 16	—	—	4.6 × 10^10^

The morphology of NPs was evaluated by SEM and it was observed that AgNpE are more spherical than AgNpR (Fig. 1), probably due to the differences in chemical composition of each reducing agent and/or due to the distinct interactions between the biomolecules existing in the different parts extracted from the plant. In addition, AgNPs did not present aggregates, and the size distribution given by the histogram showed that AgNpR presents a larger population in relation to AgNpE.

### Toxicity evaluation using *in vitro* assays with different cell lines

**Figure 2:**
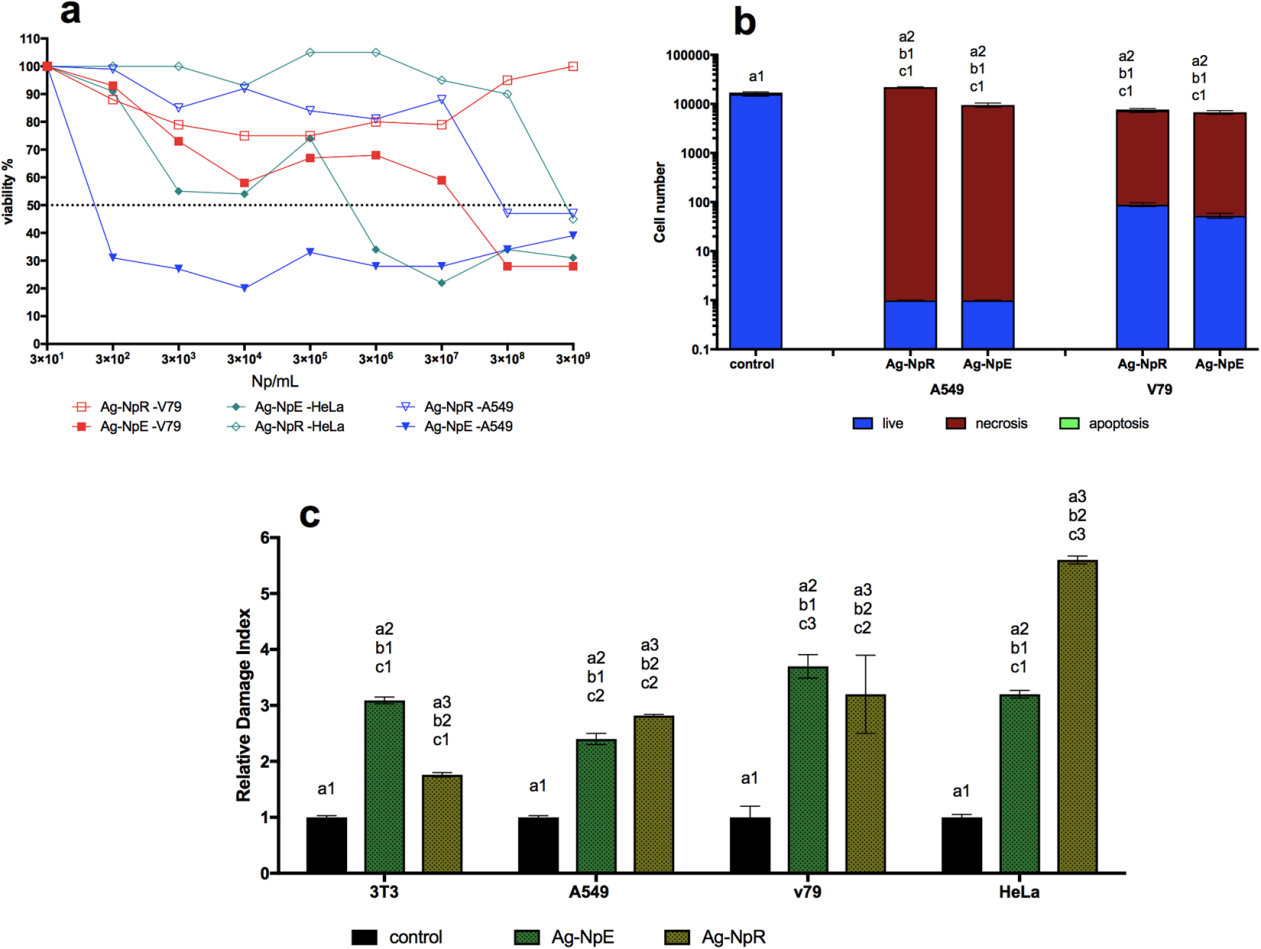
*In vitro* evaluation of toxicity. (**a**) Cell viability using the MTT test. (**b**) Imaging cytometry. (**c**) Analysis of DNA damage (comet tests). The same numbers indicate similarity and different numbers indicate statistically significant difference (p < 0.05).

Viability assays through mitochondrial metabolism performed using the MTT salt showed that AgNpR was less cytotoxic than AgNpE towards all the cell lines (Fig. 2a). The IC_50_ values obtained for A549, HeLa and V79 cell lines exposed to AgNpE were 3.75 × 10^2^, 2.78 × 10^6^, and 1.38 × 10^8^ NPs.mL^−1^, respectively. None of the nanoparticles presented cytotoxicity towards HaCat. Imaging cytometry evaluation of the effects of the nanoparticles at a concentration of 3 × 10^8^ NPs.mL^−1^ on V79 and A549 showed death by necrosis of 98% of the cells, with V79 being slightly more resistant than A549 (Fig. 2b).

In addition to the effects of nanoparticles on the functions and survival of cells, it is important to consider possible damage to the genetic material, so evaluation of genotoxicity and DNA damage was performed using the same concentrations employed in the apoptosis and necrosis assays (3 × 10^8^ NPs.mL^−1^) (Fig. 2c).

The greater potential cytotoxicity of AgNpE, compared to AgNpR, could have been due to the smaller size of the particles, as suggested previously^50–52^. Another possible contributing factor was that the biogenic AgNPs were synthesized using different solutions (leaf extract and root infusion). As discussed previously^53^, different types of biogenic nanoparticles have their own unique characteristics, and the potential toxicity and activity can be affected by the presence of coatings on the nanoparticles.

The types of cells employed could also have contributed to the observed differences, since it has been found that tumor cells present greater sensitivity to AgNPs^54^. Therefore, the results obtained using the HeLa and A549 tumor cell lines suggested that the AgNpE nanoparticles could possibly be used to combat tumor cells, supporting other studies which indicated that biogenic AgNPs might be one option for the treatment of diseases such as cancer^55–57^.

Although the comet results revealed DNA damage, which could be indicative of future death by apoptosis, this did not occur, and necrosis appeared to be the main cause of cell death (Fig. 2b). The results concerning necrosis are important, because many tumor cells possess mechanisms of resistance to apoptosis, so these nanoparticles could offer an alternative in the fight against tumor cells^58–61^.

As found for the other cell lines, the A549 and HeLa cells exhibited greater numbers of DNA breaks when exposed to AgNpR, compared to exposure to AgNpE. The viability results showed that these same cells (A549 and HeLa) presented cell death with low IC_50_ values when treated with AgNpE, so it is possible that the cells died and for this reason it was not possible to detect DNA damage.

### Evaluation of antimicrobial potential using minimum inhibitory concentration (MIC) assays

The MIC analyses showed that at concentrations of 10^6^ NPs.mL^−1^, both AgNpE and AgNpR were inhibitory towards *E*. *coli*, *S*. *aureus*, and *C*. *albicans*, while *E*. *coli* was inhibited by the AgNpR nanoparticles at 10^5^ NPs.mL^−1^. These results were in agreement with other studies demonstrating the bactericidal potential of these nanomaterials^23,62,63^. Evaluation of the pure extract of *Althaea officinalis* revealed no bactericidal or fungicidal activity, as found previously by Shah^64^.

The activity of AgNPs is similar to that of silver ions, acting at different sites, rather than according to a specific pathway, with the main mechanisms being the generation of reactive oxygen species, enzymatic inhibition, damage to the genetic material, and disruption of the mechanisms of protein synthesis^65–68^.

### Ecotoxicity evaluation using *Allium cepa*

**Figure 3:**
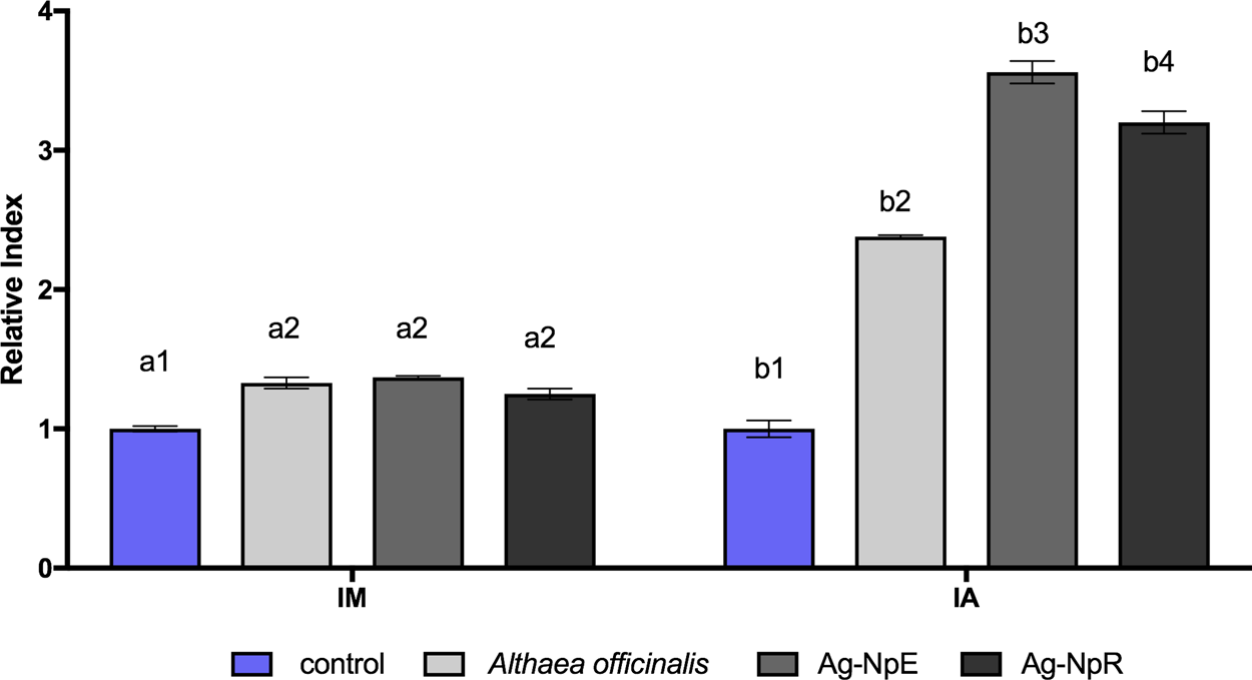
*Allium cepa* analyses performed with AgNpE, AgNpR, and *Althaea officinalis* extract. Exposure for 24 h. MI: mitotic index; AI: alteration index. The same numbers above the columns indicate similarity and different numbers indicate significant difference (p < 0.05).

The exposure of *Allium cepa* to both AgNpE and AgNpR at a concentration of 3 × 10^8^ NPs.mL^−1^ resulted in mitotic indexes increase (Fig. 3), however there was no difference between the two exposures. Similar findings were reported by Lima *et al*.^69^, for biogenic nanoparticles based on the fungus *Fusarium oxysporum*.

Both AgNpE and AgNpR showed higher aberration indexes, with AgNpE being slightly more toxic than AgNpR.

### Ecotoxicity evaluation using zebrafish

**Figure 4:**
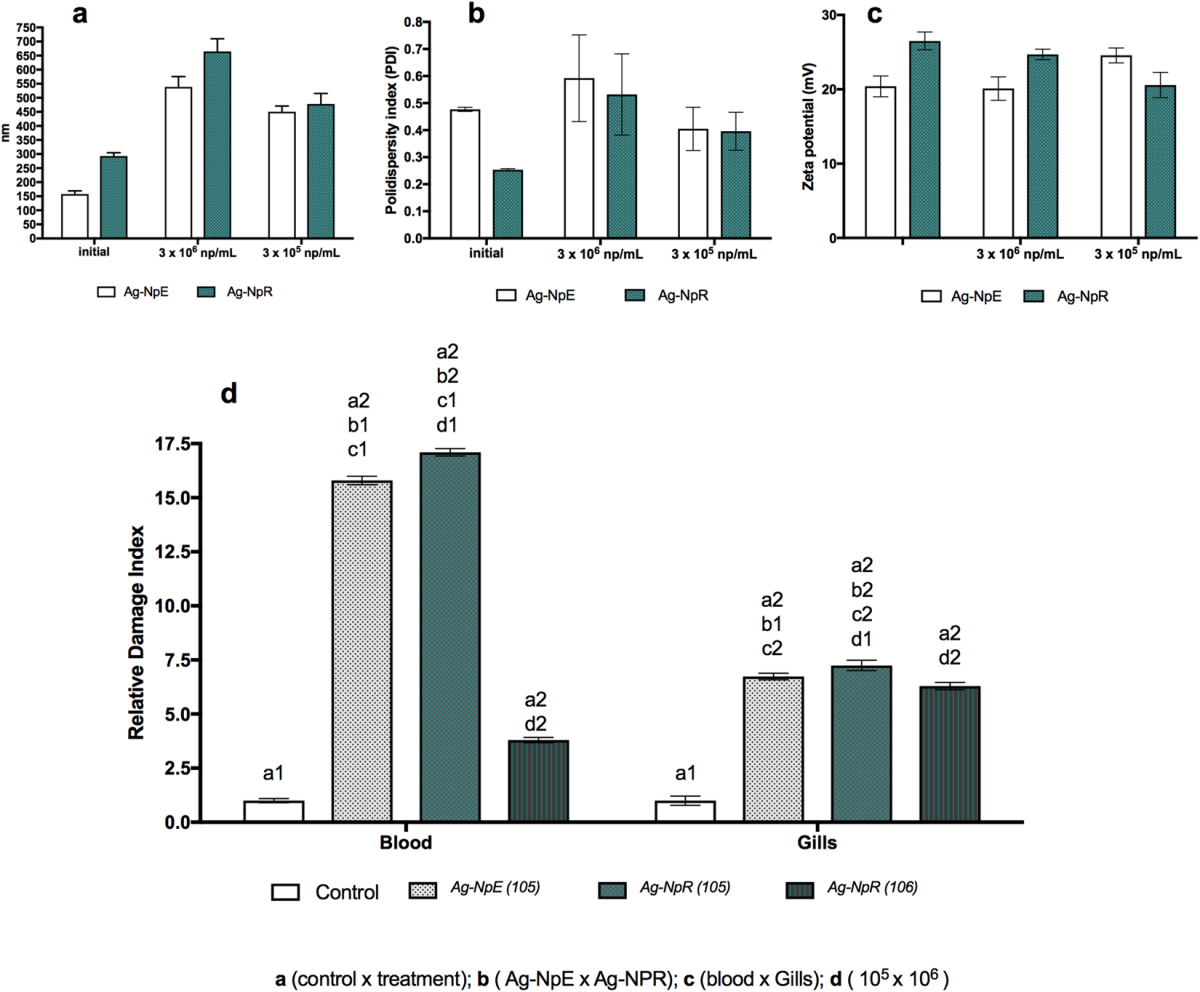
DLS analyses of the nanoparticles after exposure of the fish for 72 h. (**a**) Particle size; (**b**) polydispersity; (**c**) zeta potential; (**d**) comet analyses using blood and gill cells.

Determination of the physicochemical characteristics of the nanoparticles after the fish exposure showed diameter increase, indicating agglomeration (Fig. 4a), which can be due to interaction of the nanoparticles with the environment. According to Yin *et al*.^70^, water chemistry has a major impact on the aggregation of AgNPs, due to the combined effects of organic materials and electrolytes.

Another factor that influences nanoparticle characteristics is the presence of light, which can alter the particle morphology and increase sedimentation rates. Metabolic factors involving the fish should also be considered, since these could have modified the nanoparticles. The diameter increase may result from oxidation of the nanoparticles in the aquatic environment^71^, however it may also be a positive factor, since large aggregates tend not to affect the cells of organisms.

Exposure of zebrafish to AgNpE and AgNpR at 3 × 10^8^ and 3 × 10^7^ NPs.mL^−1^ resulted in death of the fish after 24 h, indicating high toxicity. At 3 × 10^6^ NPs.mL^−1^, the exposure of the fish to AgNpE led to death, while animals exposed to AgNpR presented greater damage in the gill cells through comet assay (Fig. 4d).

In contrast to the effects observed at the 10-fold higher concentration, exposure of the fish to AgNpE and AgNpR at 3 × 10^5^ NPs.mL^−1^ resulted in a greater number of DNA damage in the blood cells, compared to the gill cells, suggesting that a lower availability of nanoparticles reduced their accumulation in the gills facilitating the entry of nanoparticles into the organism, reaching blood cells.

It has been shown that nanoparticles can generate oxidative stress in fish, which typically leads to apoptosis, and that the Ag^+^ ions released can cause rupture damage in the system, resulting in interaction with membrane proteins and disturbance of cellular homeostasis^72–74^. Osborne *et al*.^52^ found nanoparticles accumulated in the gills and intestines, which resulted in increased production of mucus in the attempt to cleanse the organs, consequently hindering respiration and leading to death.

**Figure 5:**
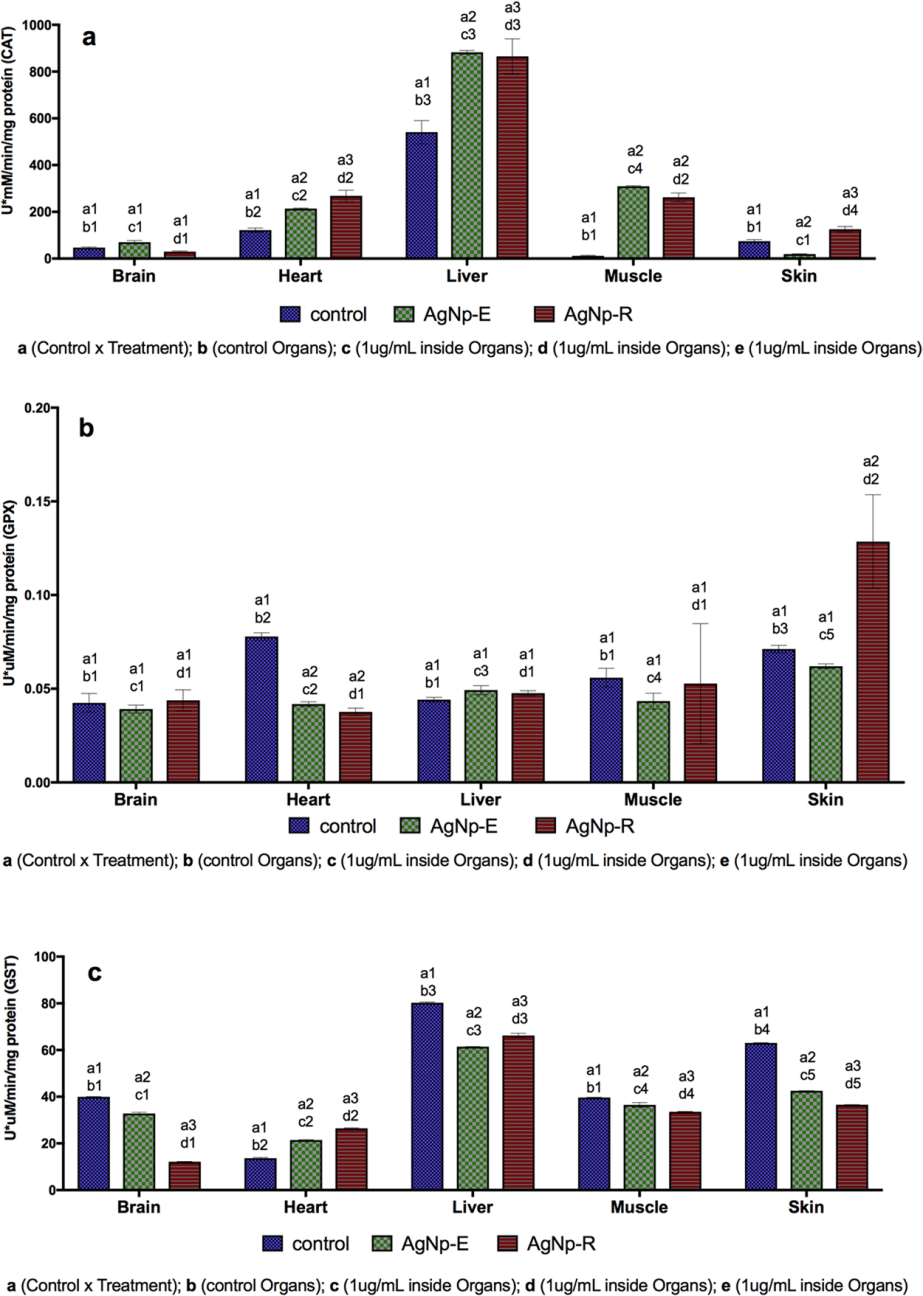
Biochemical analyses using the brain, heart, liver, muscle, and skin of zebrafish exposed to the biogenic nanoparticles. (**a**) Catalase; (**b**) Glutathione peroxidase; (**c**) Glutathione-S-transferase. The same numbers above the columns indicate similarity, while different numbers indicate significant difference (p ≤ 0.05).

The biochemical analyses showed that CAT activity increased in the heart, liver, muscle, and skin (Fig. 5a). As expected, the highest activity occurred in the liver, indicating that CAT redox system was activated following the exposure. The brain showed no significant alteration, although catalase levels in this organ are normally extremely low (Fig. 5a).

The GPx analyses showed that in most of the organs, the activity of this enzyme remained stable for both AgNpE and AgNpR, except in the skin of fish exposed to AgNpR, where there was an increase of its activity, and in the heart where a decrease was observed for both the nanoparticles (Fig. 5b).

The GST activity showed changes in all the organs, with decrease in the brain, muscle, skin, and liver (Fig. 5c). GST consists of a group of enzymes that are widely distributed in the tissues, which catalyze the conjugation of GSH and compounds with reactive electrophilic groups (including xenobiotics such as metals and pesticides). The decreased GST activities in these tissues could therefore have been related to lower levels of GSH.

Catalase (CAT) is responsible for reduction of hydrogen peroxide, so an increase of its activity in tissues can be indicative of oxidative stress. In the liver, it may reflect the presence of high levels of H_2_O_2_, associated with the accumulation and detoxification of various environmental pollutants. Oxidative stress is mostly controlled by means of CAT and superoxide dismutase (SOD) pathways. SOD and CAT respond rapidly during oxidative stress, as found in the marine gastropod *Onchidium struma* exposed to copper^75^.

Several studies have reported the exposure of aquatic organisms to nanoparticles. Choi *et al*.^76^ observed a significant decrease in CAT activity in the tissues of zebrafish exposed to commercial AgNPs, however, McCarthy *et al*.^77^ and Buffet *et al*.^78^ obtained results similar to those found in the present study by using other species of aquatic animals (*Crassostrea virginica*, *Hediste diversicolor* and *Scrobicularia plana*).

The enzyme GPx removes H_2_O_2_, which is a precursor of reactive oxygen species (ROS), hence protecting the cell membrane against lipid peroxidation. Decreased GPx activity in the heart could be a sign of toxicity associated with the production of ROS induced by the presence of nanoparticles. An explanation for this decrease/inhibition could be related to a shortage of GSH in the organ. CAT and GSH act in the reduction of H_2_O_2_, suggesting that this mechanism was activated due to the formation of large amounts of this compound, since the amount of GPx is dependent on the GSH substrate, whose function is to protect cells from the free radicals produced by oxidation.

The levels of reduced glutathione (GSH) are mainly determined by equilibrium among GSH synthesis, conjugation by glutathione S-transferase (GST), oxidation by non-enzymatic mechanisms or by GPx, and reduction of oxidized glutathione (GSSG) to GSH by glutathione reductase (GR)^79^. Similar results were obtained by Masoud *et al*.^80^ for continuous exposure of organisms to AgNPs, while other studies have found the opposite effect, with decreases of GPx activity in tilapia exposed to AgNPs at concentrations of 2 and 4 mg.L^−1^ for 15 days^81^.

Increases in GST levels are directly linked to the processes of detoxification in organisms^82^, and in the present work, this behavior was only observed in the heart. The increased GST activity in the heart could indicate that GSH was used by this organ in conjugation catalyzed by GST, leading to a decrease in GSH due to GPx activity. However, decreases in GST have been reported in organisms exposed to pesticides, as in the work of Sillapawattana *et al*.^83^, where arthropods were exposed to nanoparticles and the level of GST decreased at higher contaminant concentrations. Arle *et al*.^84^ reported the same behavior in zebrafish embryos exposed to AgNPs.

Increased activity of GST in the liver has been reported previously in studies of fish^85^ and could indicate an attempt by the organism to adapt, since it probably reduces the possibility of compounds binding to other macromolecules such as DNA. GST is a scavenger of hydroxyl radicals, and its lack can lead to disastrous consequences in organisms, such as lysis of erythrocytes, as described in *Adamussium colbecki* by Cogo *et al*.^86^.

Although there were no major changes in GST levels, the comet assays showed increased DNA damage in zebrafish blood cells exposed to 3 × 10^5^ NPs.mL^−1^ (Fig. 5c), highlighting the importance of this enzyme in the biotransformation of compounds. Its activity could potentially be employed as a good biological marker of contamination by metallic nanoparticles.

Bacchetta *et al*.^87^ found no oxidative stress in zebrafish exposed to AgNPs. Poynton *et al*.^88^ suggested three mechanisms of toxicity of AgNPs: (i) toxicity following the release of Ag^+^ ions in aquatic environments; (ii) toxicity caused by metal ions according to independent mechanisms; and (iii) the transport into the cell of Ag^+^ ions released from nanoparticles that remain outside the cell.

In order to investigate the toxicity of silver nanoparticles in aquatic systems, some studies using algae as target organism were performed. Zhang *et al*.^89,90^ have shown that silver nanomaterials could induce toxicity being this cellular toxicity caused by the modulation of reactive oxygen species (ROS) in cells as a result of the faster release of Ag species, however these nanoparticles were not biogenic.

## Conclusions

*Althaea officinalis* acts as an effective reducing and stabilizing agent for the biogenic synthesis of silver nanoparticles, which present adequate physicochemical characteristics. Even though this new nanomaterial presents potential for the control of microrganisms and tumoral cells, it is therefore necessary to consider the possible toxicity of these formulations when in direct contact with organisms or released in environment. AgNpE was slightly more toxic than AgNpR, and both materials exhibited physicochemical changes during exposure assays. The results demonstrated the importance of performing toxicological and ecotoxicological evaluations of new nanoparticles, since although they may provide the desired bactericidal effect, their toxicity could threaten the survival of organisms.

The current study highlighted the possible differences in the response of tissues to silver nanoparticles exposures, depending the biogenic AgNPs species and their concentrations. Silver nanoparticles caused oxidative stress followed by the increase in CAT and decrease in GPx and GST even in the lower concentration and in almost all tissues. In this circumstance, it seems to reflect an aggravation status due to reduced cell protection ability to protect fish against ROS under stress conditions caused by these nanoparticles. The findings show that the enzymes associated with biotransformation and detoxification can be used as biomarkers in zebrafish exposed to contamination by nanoparticles.
